# Supplementary material for: Endophilin-A coordinates priming and fusion of neurosecretory vesicles via intersectin
Source: Nat Commun. 2020 Mar 9;11:1266. doi: 10.1038/s41467-020-14993-8 (PMC7062783; doi:10.1038/s41467-020-14993-8)
Supplement: Supplementary file 1 — Supplementary Information [file 41467_2020_14993_MOESM1_ESM.pdf]

Supplementary Information

Endophilin-A coordinates priming and fusion of neurosecretory vesicles via intersectin

Sindhuja Gowrisankaran[1], Sébastien Houy[2,*], Johanna G. Peña del Castillo[1,*], Vicky Steubler[1,*], Monika Gelker[1], Jana Kroll[1], Paulo S. Pinheiro[2,3], Dirk Schwitters[1], Nils Halbsgut[1], Arndt Pechstein[4], Jan R.T. van Weering[5], Tanja Maritzen[4], Volker Haucke[4], Nuno Raimundo[6], Jakob B. Sørensen[2,#], Ira Milosevic[1,#]

Supplementary Methods

Purification of synaptic vesicles and LDCVs

Synaptic vesicles were purified as in Farsi et al. (2018) [1]. The size of murine adrenal gland at P0 is ~0.5-1mm in diameter, with the chromaffin cell containing adrenal medulla constituting only a fraction of the gland tissue. In addition, endophilin TKO mice are lethal at birth and come in a 1:4 ratio, making this task even harder. Therefore, LDCVs could not be purified from newborn mice due to technical limitations. Instead, LDCVs purified from the medulla of two bovine adrenal glands were used[2]. Protein concentration of SV and LDCV samples was determined using Pierce BCA Protein Assay Kit and following the manufacturer's instructions.

Real-time quantitative PCR

RNA extraction and purification were performed as in Fernandez-Mosquera et al. (2019) [3]. Each 8 µl reaction in a 96 well plate contained 4 µl cDNA, 0,2 µl dilutions of each primer (from 25 µM stock), and 3.6 µl iTaq Universal SYBR Green Supermix (Bio-Rad, 1725120). The qPCR was performed on (Thermocycler UNO II, Biometra) and data were analyzed by the ΔΔCT method, using hypoxanthine-guanine phosphoribosyltransferase (HPRT) as a reference gene.

pHluorin assay in mouse chromaffin cells

Primary cultured mouse adrenal chromaffin cells were infected with lentivirus expressing synaptotagmin-1-pHluorin or synaptotagmin-7-pHluorin (both plasmids were a gift of Dr. B. Tawfik, University of Copenhagen) for ~36 h. Live Syt-1-pHluorin or Syt-7-pHluorin cells were imaged using the spinning disk confocal setup (Perkin Elmer/Nikon/Volocity) with temperature control unit (kept at 37°C) and custom-built imaging chamber. Cells were maintained in the Tyrode buffer before 59 mM KCl was added for stimulating the cells. Images were analyzed using ImageJ as described in Rao et al. (2017) [4], and exocytic sites were determined primarily based on the fluorescence increase after fusion of secretory vesicles with the plasma membrane due to the change in pH.

Detection of actin in chromaffin cells

Adrenal chromaffin cells on poly-L-lysine-coated glass coverslips were treated for immunostainings as described earlier. For the F-actin analysis, the cells were incubated for 30 min with phalloidin-Texas Red dye for staining F-actin (1 U/cs; 200U/mL stock dissolved in methanol), washed, stained with DAPI and mounted using Mowiol-488® mounting medium. The

images were acquired using Zeiss LSM 800 laser scanning confocal microscope. The actin staining was analyzed by ImageJ PLASMaCC macro reported in Kurps et al. (2014) [5].

Supplementary Figures

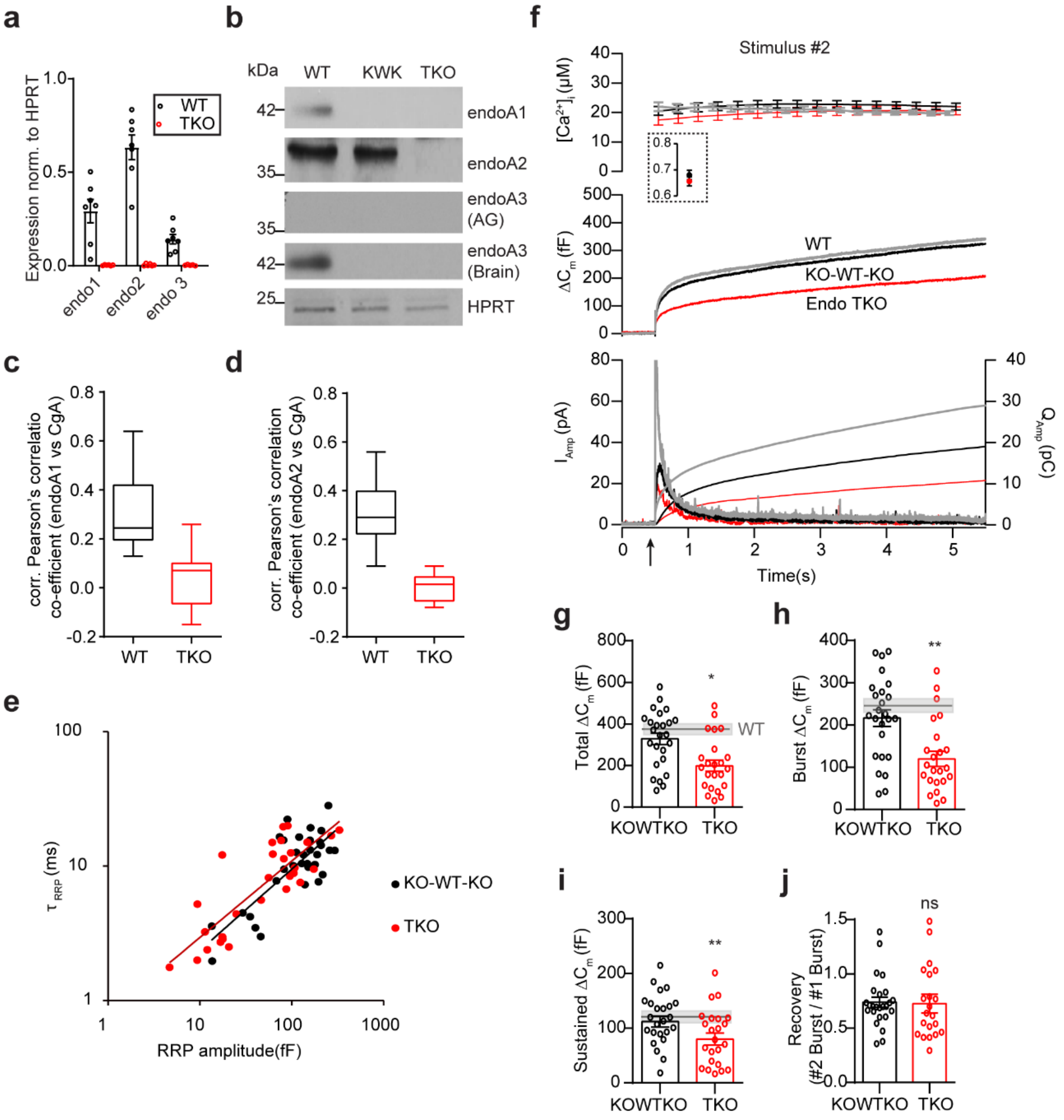

Supplementary Figure 1 (related to Figure 1)**. Endophilins are present in adrenal chromaffin cells and on LDCVs. Lack of endophilins reduces exocytosis in mouse chromaffin cells.** (a) Real-time PCR for endophilin 1, 2 and 3 performed on RNA isolated from adrenal medulla showed the presence of all three endophilins' mRNAs (N=3, mice WT (7) and TKO (6) for endophilin 1 and 2; N=3, mice WT (7) and TKO (5) for endophilin 3). (b) Representative Western blot analysis of adrenal gland lysate blotted with anti-endophilin 1, 2 and 3 antibodies. Endophilin

3 could not be detected in the adrenal gland homogenate (~40 µg), although it could be detected in the same amount of WT brain sample. Blot was selected from one out of three experiments yielding almost identical results. (c-d) Colocalization analysis of CgA with endophilin 1 (N=3, 4 mice: WT (42 cells) and 3 mice: TKO (17 cells – note that most TKO cells did not have any detectable endophilin 1 signal)), or endophilin 2 (N=3, WT 4 mice (40 cells) and TKO 3 mice (14 cells - note that most TKO cells did not have any detectable endophilin 1 signal)) respectively, was performed in WT and endophilin TKO cells as detailed in Methods and plotted as mean (central line) and min to max whiskers (note that the accidental colocalization was subtracted given the high abundance of both signals, resulting in negative correlation in some endophilin TKO cells). (e) Double-log plot of time vs. the pool size of the RRP showed correlation and suggested that the faster fusion kinetics and lower amplitude may not be two phenotypes, but one (data from Figure 1 m-o). (f-i) Exocytosis induced by a second stimulus (elicited 100s after the first stimulus) showed a similar reduction in total vesicle exocytosed as well as burst and sustained components of the release (the smaller exocytic responses prevented exponential kinetic analysis). Panels in (f) are arranged as detailed in Figure 1I (KOWTKO: N=4, 6 mice (32) and TKO: N=4, 6 mice (29). (j) Measure of recovery, calculated as the ratio of burst secretion of the second over the first stimulus was not significantly altered. KOWTKO: N=4, 6 mice (32) and TKO: N=4, 6 mice (29). Error bars denote SEM. Unpaired two-sided t-test, *p<0.05; **p<0.01, ns not significant. N=number of independent replicates.

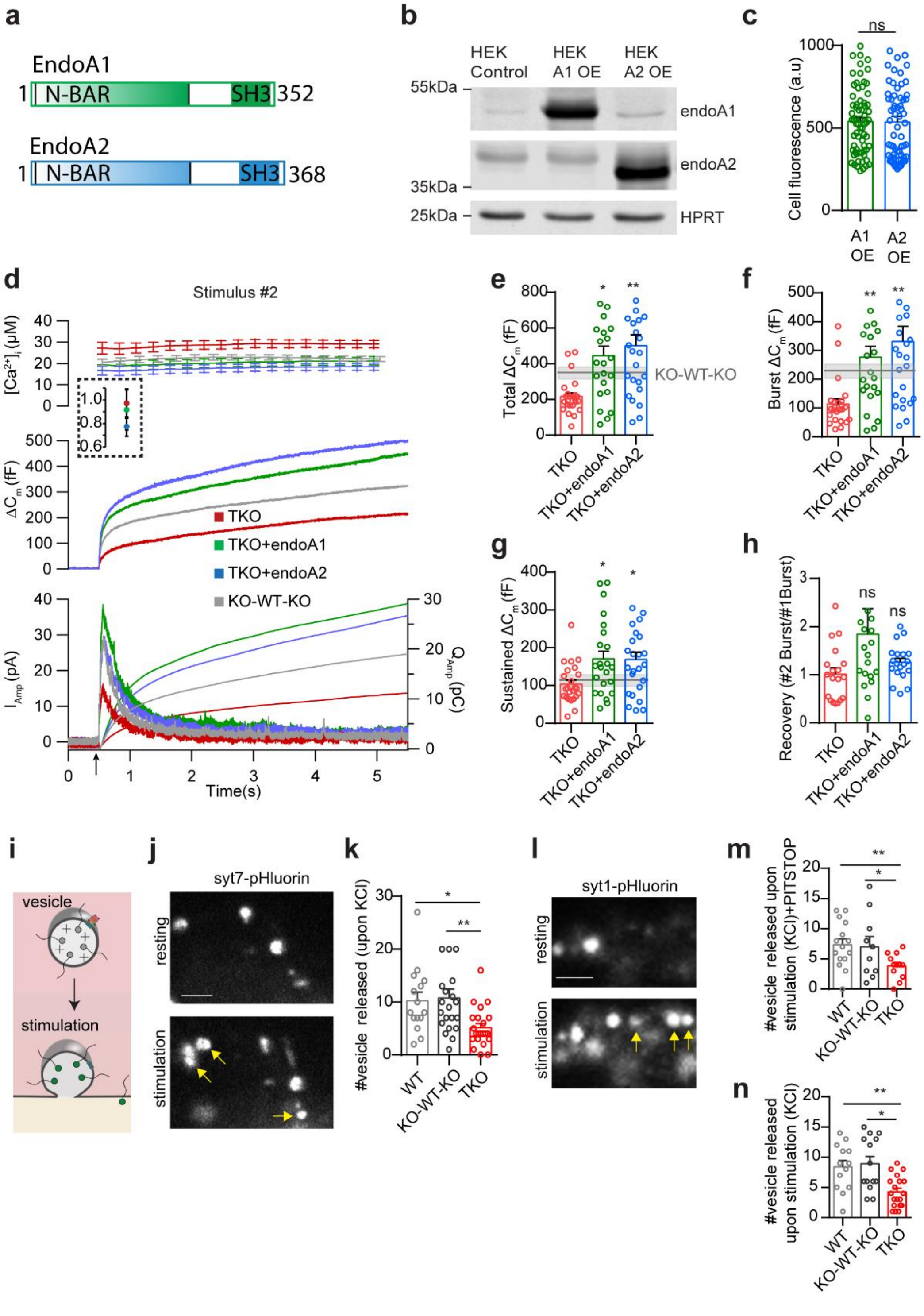

Supplementary Figure 2 (related to Figure 1, 2)**. Expression of endophilin 1 and endophilin 2 rescued exocytosis defects in endophilin TKO cells.**

(a) Schematic describing the domain structure of endophilin 1 and endophilin 2 expressed in the subsequent panels. (b) Verification of lentiviral expression system for endophilin 1 and endophilin

2 in HEK293 cells (representative blot). Three independent experiments were performed: each time a new set of HEK293 cells was transfected as indicated, collected and tested by Western blot. (c) EGFP fluorescence intensities measured from TKO cells expressing endophilin 1 and endophilin 2 using bicistronic lentiviral systems were comparable (N=3 independent experiment, A1 OE (68 cells) and A2 OE (67 cells)). (d-e) Expression of endophilin 1 and endophilin 2 rescued the exocytic defects seen in endophilin TKO cells (stimulus #2). Panel arranged as in Figure 1I, with 3 groups: endophilin TKO (red traces; N=4, 4 mice (24 cells)), endophilin TKO + endophilin 1 (green traces; N=4, 4 mice (23 cells)) and endophilin TKO + endophilin 2 (blue traces; N=4, 4 mice (25 cells)). Endophilin KOWTKO data from Figure S1F (gray trace) were superimposed. Note that endophilin 1 as well as endophilin 2 could rescue exocytosis. (f-g) Burst and sustained components were rescued upon expression of endophilin 1 and 2, respectively. (h) Recovery, calculated as the ratio of burst secretion of the second over the first stimulus, was not significantly altered (N=4, 4 mice (TKO=19 cells, TKO+endophilin 18 cells, TKO+endophilin 19 cells). (i-n) Vesicle release upon stimulation measured by pHluorin-based imaging assay (schematic in i). (j) Exemplary images of selected region-of-interest from chromaffin cell expressing syt-7-pHluorin in the resting vs. stimulated (59 mM KCl) condition. (k) Endophilin TKO cells expressing syt-7-pHluorin showed a lower number of fluorescent puncta that appeared upon stimulation than WT and littermate KOWTKO controls (N=3 independent experiment, 3 mice per genotype, WT (15), KOWTKO (21) and TKO (22) cells). (l) Exemplary images of selected region-of-interest from chromaffin cell expressing syt-1-pHluorin in the resting vs. stimulated (59 mM KCl) condition. (m) Release measured as in (k) in the presence of Pitstop-2 was lower in endophilin TKO cells (N=3 independent experiment, WT 3 mice (15), KOWTKO 2 mice (10) and TKO 3 mice (22) cells). (n) Secretory vesicles released upon stimulation in TKO cells were reduced (N=3 independent experiment, WT 3 mice (13), KOWTKO 2 mice (14) and TKO 3 mice (19) cells). Error bars denote SEM. Statistics were performed by one-way ANOVA with Tukey's post-hoc test, *$p<0.05$; **$p<0.01$; ns not significant. N=number of independent replicates.

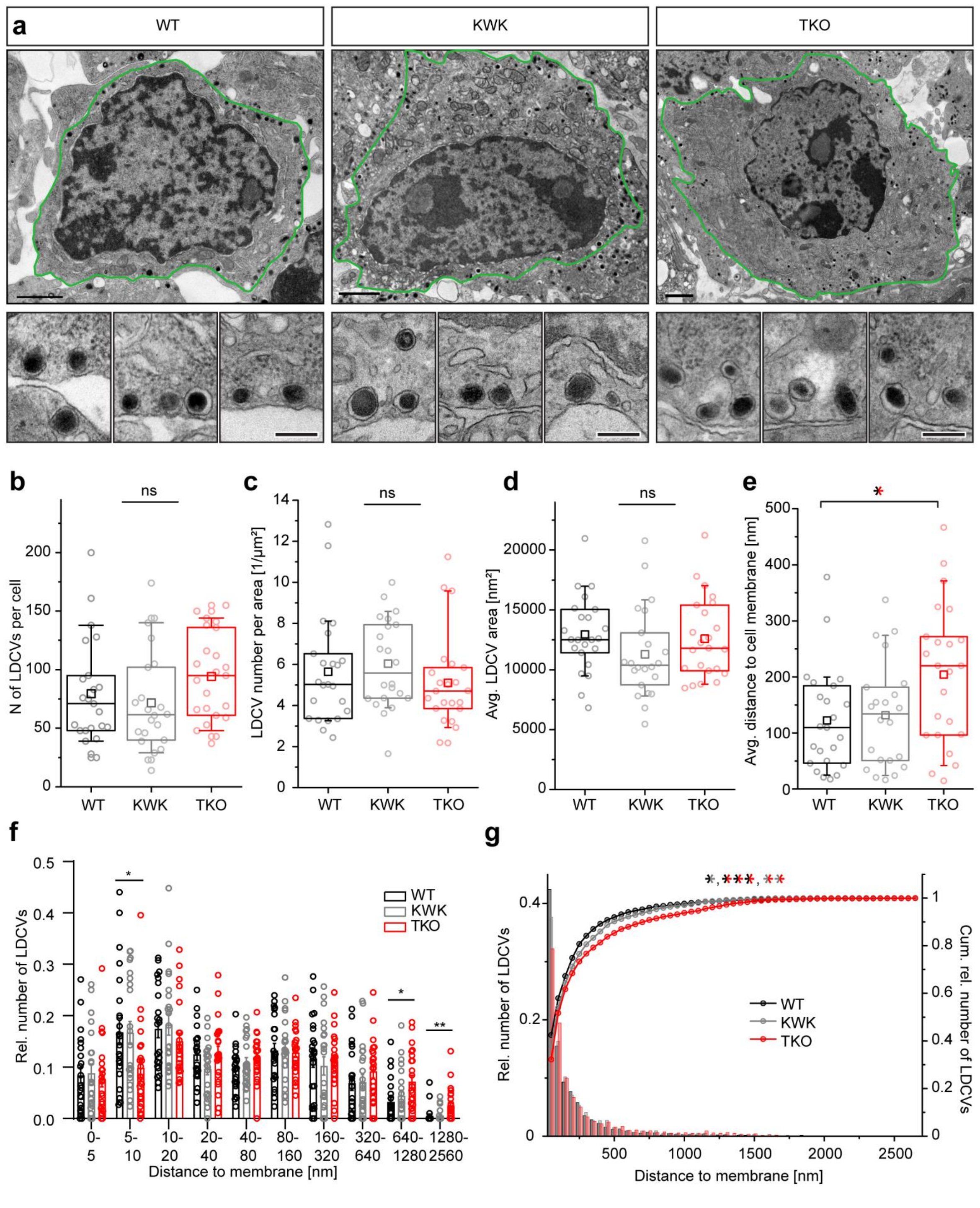

Supplementary Figure 3 (related to Figure 3). **Number and size of LDCVs were not altered in endophilin TKO chromaffin cells, but fewer LDCVs were found in the plasma membrane proximity.**

(a) Example EM images of WT, endophilin KWK (littermate control) and endophilin TKO chromaffin cells in the adrenal gland. Green line highlights one cell in the image. Note that the TKO cell is shown at lower magnification so the whole cell can be displayed. Panels below show higher magnification of LDCVs in the proximity of the plasma membrane (three examples are shown). Scale bars: 1 µm upper panels, 250 nm lower panels. (b-c) The total number of LDCVs per cell, and cell area, were unchanged between endophilin TKO and the control cells. (d) The average LDCV area was unchanged between endophilin TKO and the control cells. (e) The average distance of LDCVs from the plasma membrane was increased in cells without endophilins (one-way ANOVA after Tukey's post-hoc test, center line represents mean, whiskers indicate min. to max. in b-e). (f) Distances between the LDCV membrane and the plasma membrane, after being normalized per cell. Data shown in b-g are from 4 different animals and independent embeddings per group; WT (23 cells), KOWTKO (22 cells) and TKO (23 cells). Kolmogorov-Smirnov test with Bonferroni-correction, only significant differences are indicated. (g) Relative frequency distribution and cumulative plots produced by binning all vesicles revealed altered distribution of LDCVs in endophilin TKO cells from whole glands. Kolmogorov-Smirnov test with Bonferroni-correction. (b-g) Error bars denote SEM. $*p<0.05$, $**p<0.01$, $***p<0.001$, ns not significant.

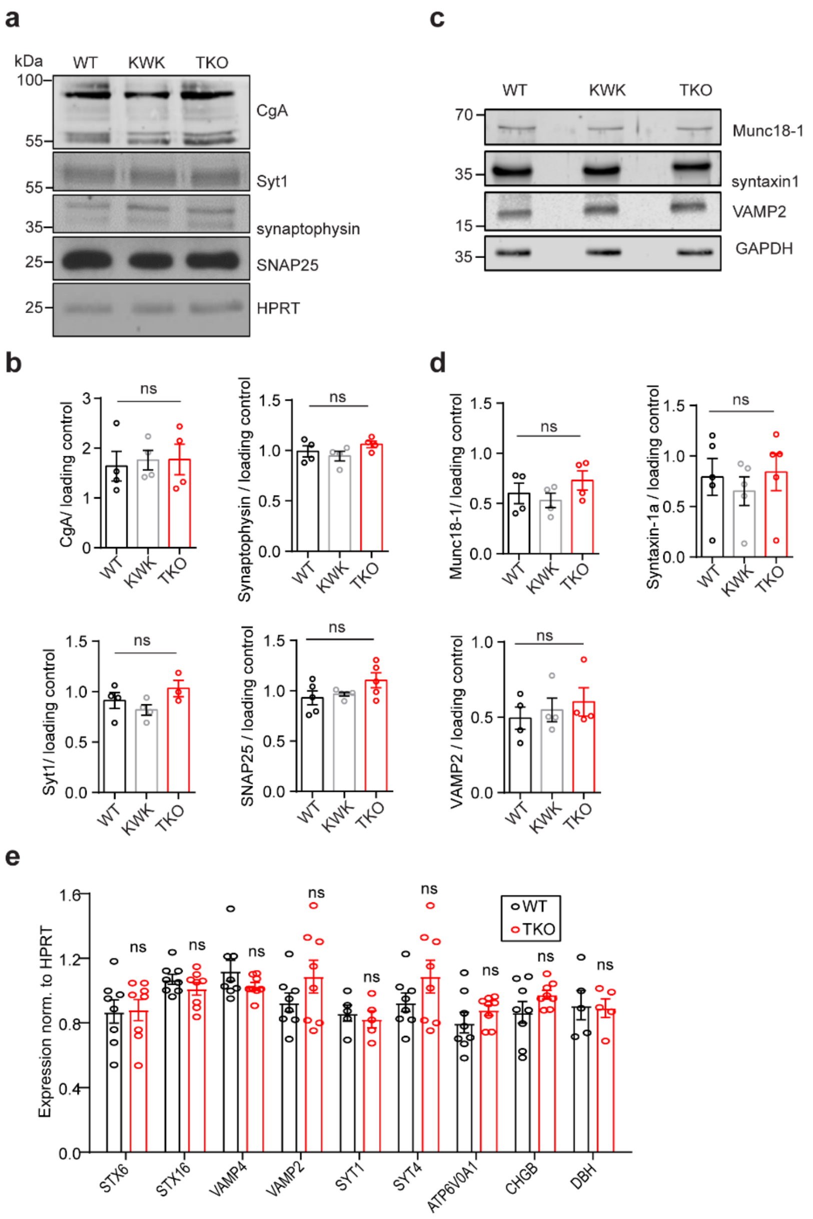

Supplementary Figure 4 (related to Figure 4). **Levels of key exocytic proteins were unaltered in mouse adrenal gland homogenates.**

(a) Representative Western blots for chromogranin-A (CgA), synaptotagmin-1 (Syt1), synaptophysin and SNAP25 showed no difference in the total protein levels between endophilin TKO cells and controls (littermate KWK and WT). (b) Quantification of Western blot data shown in A (N=4 experiments, 4 samples/genotype were analyzed, note that each sample on the blot originates from glands from 3-4 mice of the same genotype pooled together). (c-d) Western blots for Munc18-1, syntaxin-1, and VAMP2 showed no difference in the total protein levels between

endophilin TKO cells and controls (littermate KWK and WT). Quantified in (d). Munc18-1 (N=4), syntaxin-1 (N=5), VAMP2 (N=4), in each experiment 4 samples/genotype were analyzed; each sample on the blot originates from glands from 3-4 mice of the same genotype pooled together. Statistics: one-way ANOVA with Tukey's post-hoc test. (e) Real-time qPCR analysis in TKO and WT control revealed no change in the transcript levels of several key exocytic proteins (Syt1 and dopamine beta-hydroxylase/DBH: 5 animals/genotype were tested, syntaxin6/stx6, syntaxin16/stx16, VAMP2, VAMP4, synaptotagmin-4/syt4, ATP6V_oA1 and chromogranin B/CHGB: 8 animals/genotype were tested; statistics: unpaired two-sided t-test for each gene). (b,d,e) Error bars denote SEM. ns not significant. N=number of independent replicates.

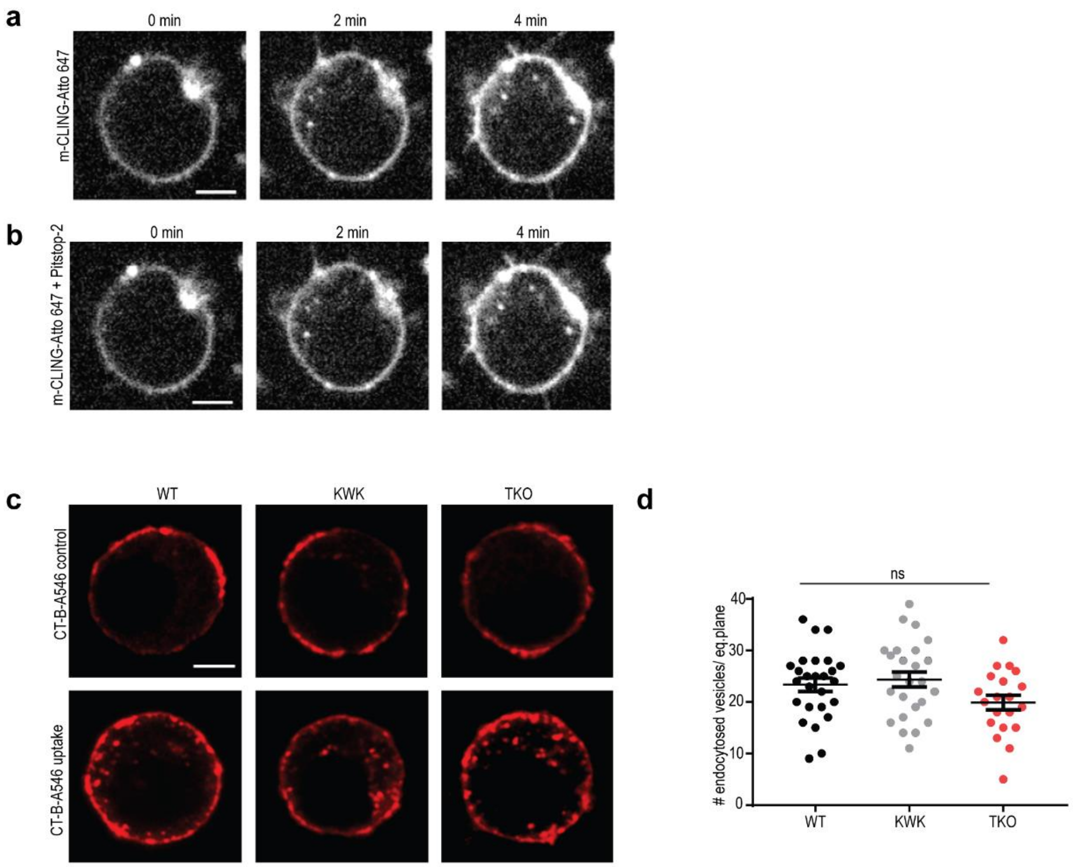

Supplementary Figure 5 (related to Figure 5). **Characterization of uptake of mCling-Atto647 and choleratoxin-B-A594 endocytic assays in adrenal chromaffin cells.** (a) WT mouse chromaffin cells were incubated with either negative control for Pitstop-2 (a), or Pitstop-2 (b) for 10 min prior to the start of the experiment. Images shown here were acquired at 30 s, 2.30 min and 4.30 min after mCling-Atto647 dye addition, and cells were stimulated (high K^+) 30 s after mCling-Atto647 dye addition (shown as 0, 2 and 4 min in the panel). Scale bar 2 µm. Considerably lower uptake of mCling-Atto647 was observed in the presence of Pitstop-2, also see Supplementary Movie 2. (c-d) Endocytic uptake in chromaffin cells was also tested using CT-B-A594 (non-toxic recombinant cholera toxin subunit-B conjugated to Alexa Fluor 594). Scale bar 2 µm. Endophilin TKO cells were compared to the littermate controls (endophilin KOWTKO) and WT. Note that uptake in the equatorial optical plane alone was analyzed in this case, unlike in whole cell as in the case of transferrin. Quantified in (d) (N=3, WT 3 mice (26 cells), KOWTKO 3 mice (25 cells) and TKO 3 mice (20 cells), one-way ANOVA with Tukey's post-hoc test. Error bars denote SEM. ns not significant. N=number of independent replicates.

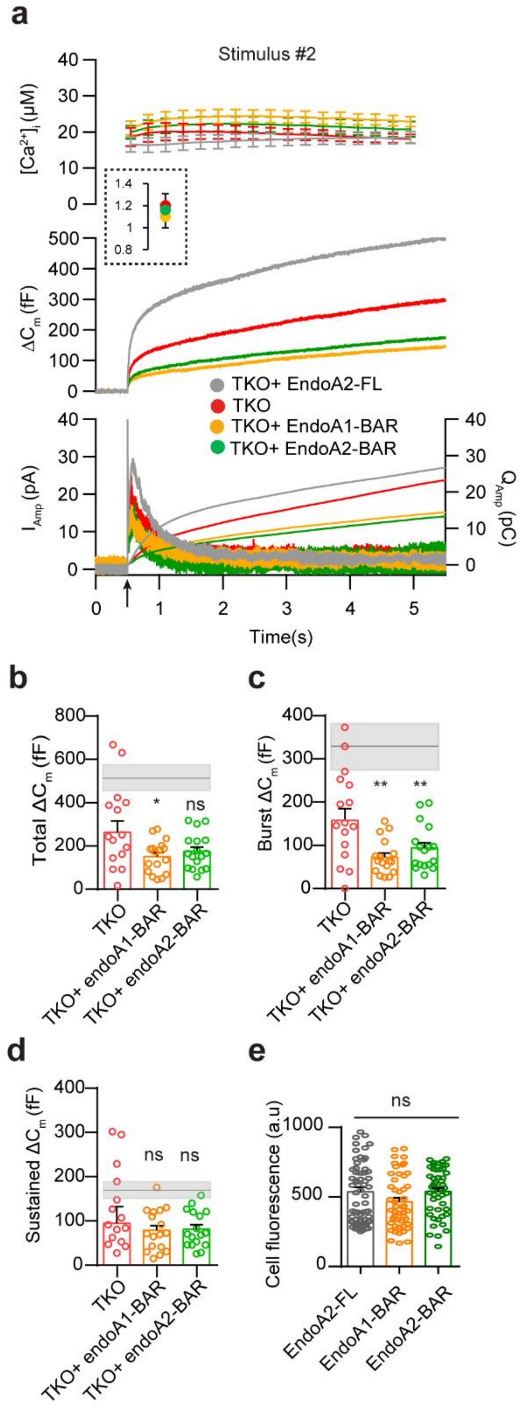

Supplementary Figure 6 (related to Figure 6)**. Endophilin 1 BAR and endophilin 2 BAR-domains are not sufficient to mediate exocytic release from chromaffin cells (second stimulation).**

(a) Exocytosis induced by calcium uncaging in endophilin TKO chromaffin cells compared to TKO cells expressing either endophilin 1 BAR or endophilin 2 BAR domain after second stimulation. Panel arranged as in Fig. 1, top: intracellular calcium level increase induced by flash photolysis at 0.5s (at arrow). The inset shows the pre-flash calcium levels. Middle: averaged traces of membrane capacitance upon Ca^{2+}-induced exocytosis. Bottom: mean amperometric current (left axis) and cumulative charge (right axis). (b-d) Quantification of changes in capacitance revealed a further reduction in different phases of release (burst and sustained) in TKO cells expressing endophilin 1 BAR or endophilin 2 BAR domain. Gray line indicates the mean of cells expressing endophilin 2 full-length protein and the shaded area indicates the SEM. (a-d) N=3, 3 mice each, TKO(16 cells), TKO+endophilin1 BAR (17 cells) and TKO+endophilin2 BAR (17 cells), one-way ANOVA with Tukey's post-hoc test. (e) EGFP fluorescence intensity measured from cells expressing endophilin 2 and the two endophilin mutants, using bicistronic lentiviral systems, were comparable (N=3, endophilin 2 expression (67 cells from 3 mice), endophilin 1-BAR (48 cells from 3 mice) and endophilin 2-BAR (47 cells from 3 mice), one-way ANOVA with Tukey's post-hoc test. Error bars denote SEM. *$p<0.05$; **$p<0.01$; ns not significant. N=number of independent replicates.

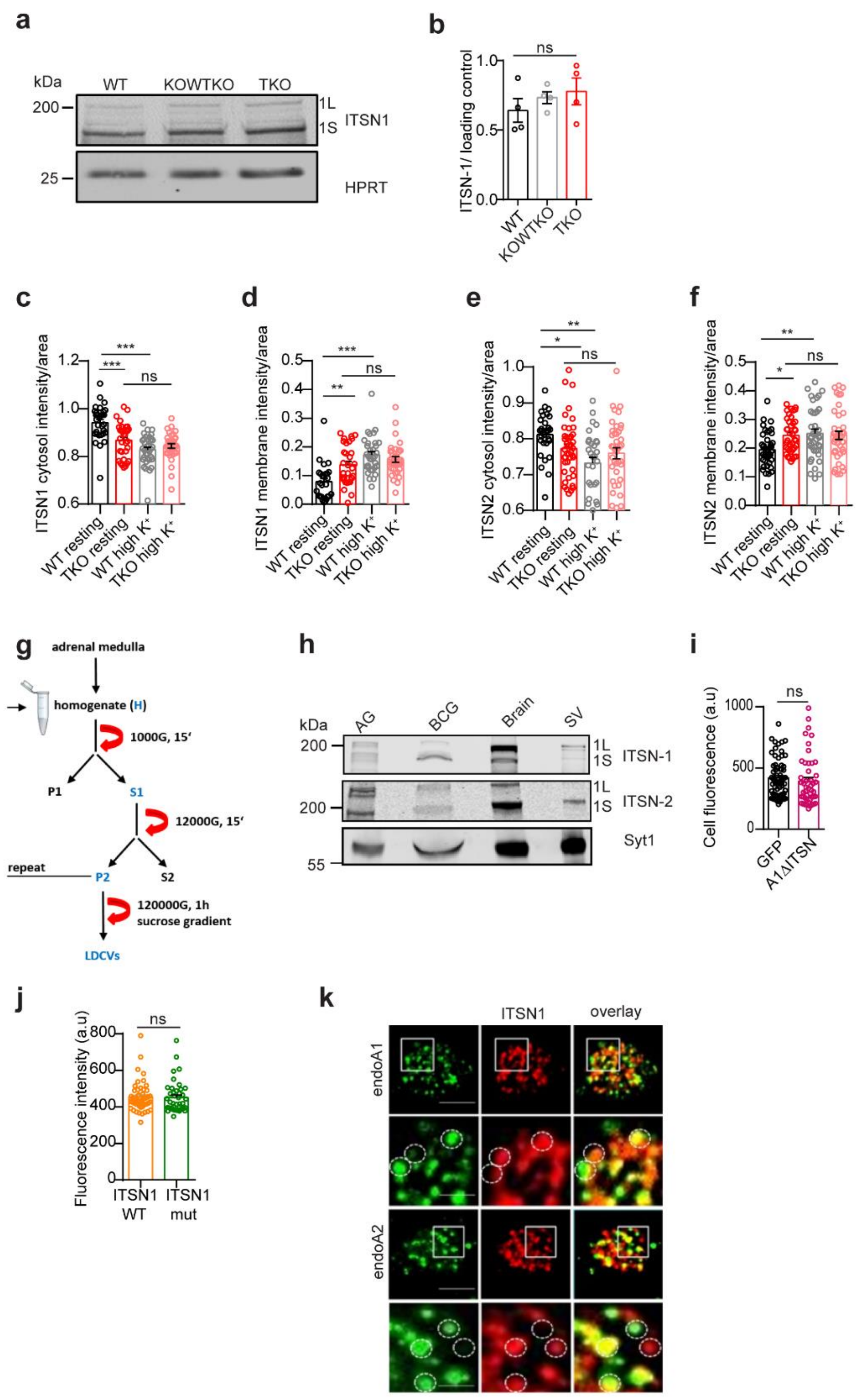

Supplementary Figure 7 (related to Figure 7)**. Levels of intersectin-1 protein were not altered in endophilin TKO cells.**

(a-b) Western blot for ITSN-1 showed no difference in the total protein levels in endophilin TKO and controls (endophilin KOWTKO and WT); quantified in (b). N=4, 4 samples/genotype were analyzed, each sample is a pool from 3-4 mice of same genotype. Statistics: One-way ANOVA. (c-d) Quantification of ITSN-1 distribution in cell cytosol (c) and near the membrane (d) at resting and stimulated (depolarization by high K^+) conditions showed altered ITSN-1 distribution in the endophilin TKO cells. (e-f) Quantification of ITSN-2 (same as ITSN-1) on cell cytosol (e) and near the membrane (f) are represented as bar graphs. Statistics: one-way ANOVA. The ratio of membrane to cytosol intensity is shown in Figure 7B and 7d (please also refer to Figure 7b and 7d for sample size). (g-h) Immunoblot analysis of LDCVs against ITSN-1 and ITSN-2 detected the presence of a small amount of ITSN-1 and ITSN-2 proteins on LDCVs and SVs (AG: Adrenal gland homogenate, BCG: purified bovine chromaffin LDCVs, brain: brain homogenate, SV: purified synaptic vesicles, Syt1 - synaptotagmin1; schematic: S - supernatant, P - pellet). Equal amount of protein is loaded in all samples shown by Western blot. Two independent experiments were done. (i) Quantification of endophilin WT and endophilin 1-E329K+S336K mutant expression (mutant that does not bind ITSN-1; used in Figure 7G-M) in chromaffin cells by fluorescence microscopy (N=3, 3 mice with GFP (48 cells) and endophilin 1-ITSN (48 cells) expression, unpaired two-sided t-test). (j) Quantification of intersectin-1 WT and intersectin-1 W949E+Y965E mutant expression (mutant that does not bind endophilin-1; used in Figure 8) in chromaffin cells by fluorescence microscopy (N=3 exp, 3 mice with ITSN WT (48 cells) and ITSN1Δendo (42 cells) expression, unpaired two-sided t-test). (k) Plasma membrane sheets from WT mouse chromaffin cells stained for endogenous endophilin 1 (up; or endophilin 2 - down) and ITSN-1. Scale bar 2 µm. Colocalization (after accidental colocalization was subtracted): 0.20±0.07 for endophilin 1 (N=2 exp, 3 mice (24 cells)), and 0.22±0.08 for endophilin A2 (N=3 exp, 3 mice (28 cells)). Error bars denote SEM. *p<0.05; **p<0.01, ***p<0.001, ns not significant. N=number of independent replicates.

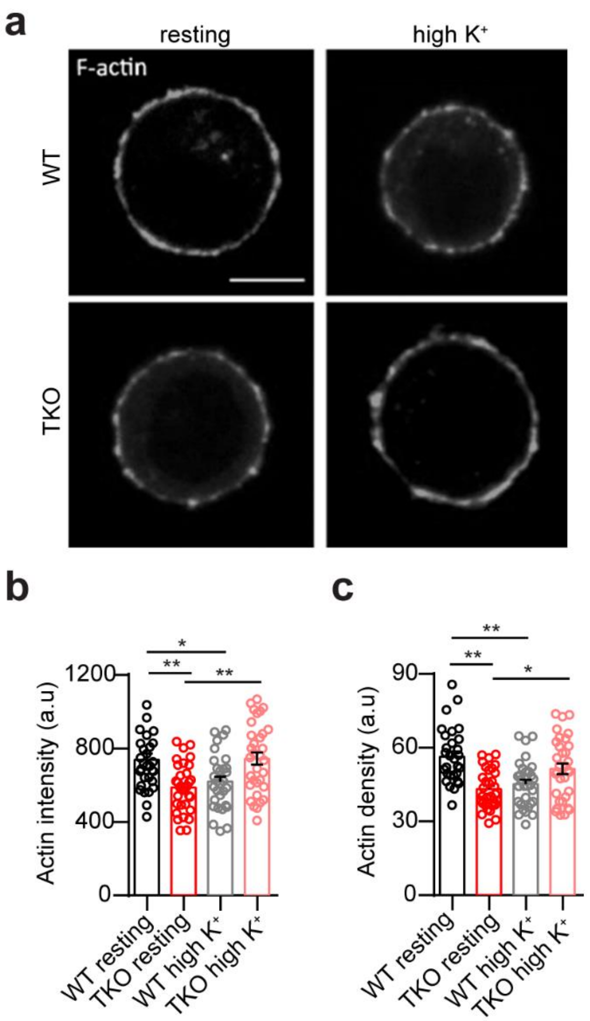

Supplementary Figure 8 (related to Figure 7 and 8)**. F-actin network is altered in the endophilin TKO cells.**

(a) Confocal image (through the equatorial plane) of WT and endophilin TKO chromaffin cells stained with phalloidin-Texas Red under resting vs. stimulated (depolarization by high K^+ solution) conditions. Note that the intensity and density of F-actin were lower in the proximity of the plasma membrane in endophilin TKO cell under resting condition. Scale bar 3 µm. (b-c) Quantification of the cortical F-actin intensity (b) and average density (c) using PLasMACC plugin in ImageJ software (N=3, 3 mice per condition, WT resting (30 cells), TKO resting (32 cells), WT high K (30 cells) and TKO high K (33 cells); one-way ANOVA with Tukey's post-hoc test, *$p<0.05$; **$p<0.01$. Error bars denote SEM.

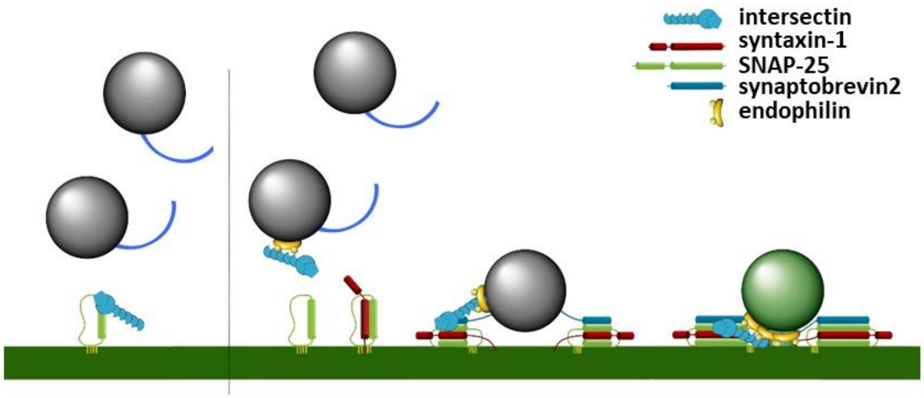

Supplementary Figure 9. Model of endophilin's and intersectin's role in exocytosis. Endophilin is present on at least some neurosecretory vesicles and has a role in vesicle recruitment, priming, and fusion. It can also regulate intersectin localization, possibly to ensure that intersectin acts only at the optimal location and time. (Left) Without endophilin, intersectin mislocalizes to the plasma membrane, so its reported role in exocytosis is likely altered in endophilin TKO cells. (Right) We propose that endophilin and intersectin act in tandem to stimulate the recruitment of secretory vesicles to the plasma membrane and their site of release, expectedly through modulation of the actin network. In addition, endophilin and intersectin may stabilize the SNARE complex between the vesicular VAMP2/synaptobrevin-2 and plasma membrane-resident SNAP25 and syntaxin-1. We also propose that once recruited to the plasma membrane, endophilin and intersectin do not dissociate after exocytosis but take part in the subsequent endocytic steps. As such, endophilin and intersectin act as a scaffold that couples exocytic and endocytic events.

In addition to data presented here, this model is supported by a number of reports [6–15].

Figure 5b

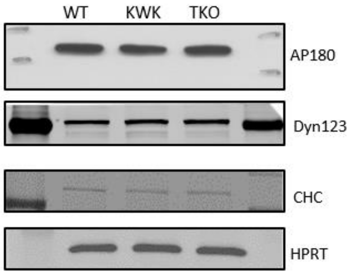

Suppl. Figure 4a

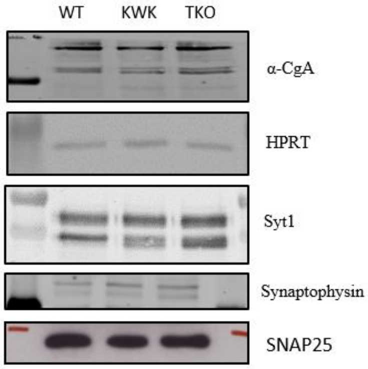

Suppl. Figure 4c

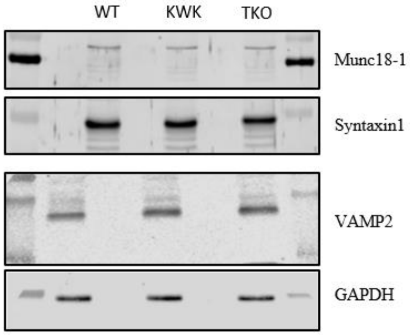

Suppl. Figure 7a

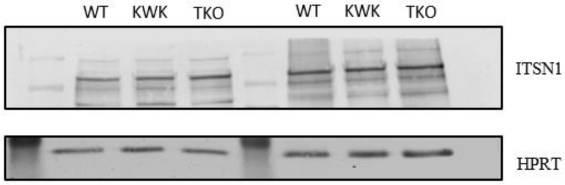

Suppl. Figure 2b

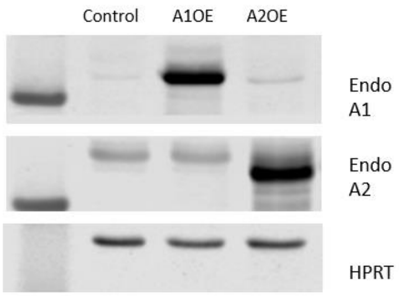

Suppl. Figure 1b

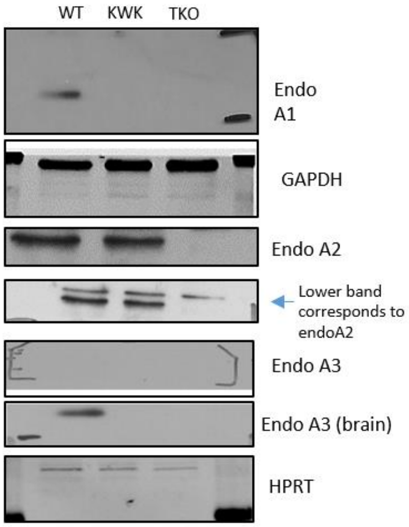

Suppl. Figure 7h

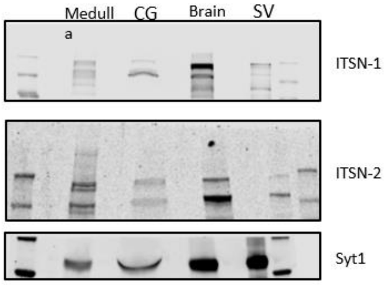

Supplementary Figure 10. Original Western blot data shown in this study.

Supplemental references

1. Farsi, Z. *et al.* Clathrin coat controls synaptic vesicle acidification by blocking vacuolar ATPase activity. *eLife* **7**, e32569 (2018).
2. Park, Y. *et al.* Controlling synaptotagmin activity by electrostatic screening. *Nature Structural & Molecular Biology* **19**, 991–997 (2012).
3. Fernández-Mosquera, L. *et al.* Acute and chronic mitochondrial respiratory chain deficiency differentially regulate lysosomal biogenesis. *Sci Rep* **7**, 45076 (2017).
4. Rao, T. C. *et al.* Synaptotagmin isoforms confer distinct activation kinetics and dynamics to chromaffin cell granules. *J Gen Physiol* **149**, 763–780 (2017).
5. Kurps, J. *et al.* Quantitative image analysis tool to study the plasma membrane localization of proteins and cortical actin in neuroendocrine cells. *Journal of Neuroscience Methods* **236**, 1–10 (2014).
6. Okamoto, M., Schoch, S. & Südhof, T. C. EHSH1/Intersectin, a Protein That Contains EH and SH3 Domains and Binds to Dynamin and SNAP-25. *Journal of Biological Chemistry* **274**, 18446–18454 (1999).
7. Malacombe, M. *et al.* Intersectin-1L nucleotide exchange factor regulates secretory granule exocytosis by activating Cdc42. *The EMBO Journal* **25**, 3494–3503 (2006).
8. Yu, Y. *et al.* Mice deficient for the chromosome 21 ortholog Itsn1 exhibit vesicle-trafficking abnormalities. *Human Molecular Genetics* **17**, 3281–3290 (2008).
9. Momboisse, F. *et al.* The Rho guanine nucleotide exchange factors Intersectin 1L and β-Pix control calcium-regulated exocytosis in neuroendocrine PC12 cells. *Cellular and Molecular Neurobiology* **30**, 1327–1333 (2010).
10. Bai, J., Hu, Z., Dittman, J. S., Pym, E. C. G. & Kaplan, J. M. Endophilin Functions as a Membrane-Bending Molecule and Is Delivered to Endocytic Zones by Exocytosis. *Cell* **143**, 430–441 (2010).

11. Milosevic, I. *et al.* Recruitment of Endophilin to Clathrin-Coated Pit Necks Is Required for Efficient Vesicle Uncoating after Fission. *Neuron* **72**, 587–601 (2011).
12. Winther, A. M. E. *et al.* The dynamin-binding domains of Dap160/intersectin affect bulk membrane retrieval in synapses. *Journal of Cell Science* **126**, 1021–1031 (2013).
13. Sakaba, T. *et al.* Fast neurotransmitter release regulated by the endocytic scaffold intersectin. *Proceedings of the National Academy of Sciences* **110**, 8266–8271 (2013).
14. Pechstein, A. *et al.* Vesicle uncoating regulated by SH 3- SH 3 domain-mediated complex formation between endophilin and intersectin at synapses. *EMBO reports* **16**, 232–239 (2014).
15. Gerth, F. *et al.* Intersectin associates with synapsin and regulates its nanoscale localization and function. *Proceedings of the National Academy of Sciences* **114**, 12057–12062 (2017).
