# Supplementary material for: Endophilin-A coordinates priming and fusion of neurosecretory vesicles via intersectin
Source: Nat Commun. 2020 Mar 9;11:1266. doi: 10.1038/s41467-020-14993-8 (PMC7062783; doi:10.1038/s41467-020-14993-8)
Supplement: Supplementary file 3 — Description of Additional Supplementary Files [file 41467_2020_14993_MOESM3_ESM.docx]

Description of Additional Supplementary Files

**Title: Supplementary Movie 1.** Representative WT mouse chromaffin cell filmed 30s after addition of mClingAtto647 dye for 12 minutes. The high potassium buffer (stimulating exocytosis and subsequently endocytosis in chromaffin cells) was added after 2 minutes. Movie speed: 10f/s.

**Title: Supplementary Movie 2.** WT mouse chromaffin cell incubated with Pitstop-2 for 10 min before mClingAtto647 dye is added. The cells are filmed 30 s after addition of mCling-Atto647 dye for 15 minutes. The high potassium buffer was added after 3 minutes. Note that the cell membrane surface area increased since endocytosis was efficiently inhibited. Movie speed: 10f/s

**Title: Supplementary Movie 3.** Representative endophilin KOWTKO mouse chromaffin cell filmed 30 s after addition of mCling-Atto647 dye for 8 min. Movie speed: 10f/s.

**Title: Supplementary Movie 4.** Representative endophilin TKO mouse chromaffin cell filmed 30 s after addition of mCling-Atto647 dye for 8 min. Movie speed: 10f/s.

**Title: Supplementary Movie 5.** Translocation of expressed ITSN-1-EGFP to the plasma membrane upon stimulation by high potassium buffer (stimulating exocytosis in chromaffin cells; KCl addition is indicated by a red label in the Movie). Movie speed: 4f/s.
