# Supplementary material for: Endophilin-A coordinates priming and fusion of neurosecretory vesicles via intersectin
Source: Nat Commun. 2020 Mar 9;11:1266. doi: 10.1038/s41467-020-14993-8 (PMC7062783; doi:10.1038/s41467-020-14993-8)
Supplement: Supplementary file 10 — Reporting Summary [file 41467_2020_14993_MOESM10_ESM.pdf]

nature research

Corresponding author(s): Dr Ira Milosevic

Reporting Summary

Nature Research wishes to improve the reproducibility of the work that we publish. This form provides structure for consistency and transparency in reporting. For further information on Nature Research policies, see Authors & Referees and the Editorial Policy Checklist.

Statistical parameters

When statistical analyses are reported, confirm that the following items are present in the relevant location (e.g. figure legend, table legend, main text, or Methods section).

n/a	Confirmed	
☐	☒	The exact sample size (*n*) for each experimental group/condition, given as a discrete number and unit of measurement
☐	☒	An indication of whether measurements were taken from distinct samples or whether the same sample was measured repeatedly
☐	☒	The statistical test(s) used AND whether they are one- or two-sided<br>*Only common tests should be described solely by name; describe more complex techniques in the Methods section.*
☒	☐	A description of all covariates tested
☐	☒	A description of any assumptions or corrections, such as tests of normality and adjustment for multiple comparisons
☐	☒	A full description of the statistics including central tendency (e.g. means) or other basic estimates (e.g. regression coefficient) AND variation (e.g. standard deviation) or associated estimates of uncertainty (e.g. confidence intervals)
☐	☒	For null hypothesis testing, the test statistic (e.g. *F*, *t*, *r*) with confidence intervals, effect sizes, degrees of freedom and *P* value noted<br>*Give P values as exact values whenever suitable.*
☒	☐	For Bayesian analysis, information on the choice of priors and Markov chain Monte Carlo settings
☒	☐	For hierarchical and complex designs, identification of the appropriate level for tests and full reporting of outcomes
☒	☐	Estimates of effect sizes (e.g. Cohen's *d*, Pearson's *r*), indicating how they were calculated
☐	☒	Clearly defined error bars<br>*State explicitly what error bars represent (e.g. SD, SE, CI)*

Our web collection on statistics for biologists may be useful.

Software and code

Policy information about availability of computer code

Data collection	Pulse/PULSEFIT (HEKA electronics), Volocity 6 (PerkinElmer), ZEN black (Carl Zeiss AG), ZEN blue (Carl Zeiss AG), Image Studio (Li-cor Bioscience), Microsoft Office – Excel (Microsoft Corporation), LightCycler 480 1.5.1.62 (Roche Life Sciences)<br>For detailed information, see manuscript.
Data analysis	Adobe Illustrator CS5.1 (Adobe Systems Inc.), Fiji/ImageJ (NIH), Igor Pro (Wavemetrics), Microsoft Office (Microsoft Corporation), Prism 6 (GraphPad), IMOD (bio3d.colorado.edu/imod), Sigma Plot 13 (Systat Software GmbH)<br>For detailed information, see manuscript.

For manuscripts utilizing custom algorithms or software that are central to the research but not yet described in published literature, software must be made available to editors/reviewers upon request. We strongly encourage code deposition in a community repository (e.g. GitHub). See the Nature Research guidelines for submitting code & software for further information.

Data

Policy information about availability of data

All manuscripts must include a data availability statement. This statement should provide the following information, where applicable:

- Accession codes, unique identifiers, or web links for publicly available datasets
- A list of figures that have associated raw data
- A description of any restrictions on data availability

All raw data that support the findings and figures included in this manuscript are available from the corresponding author upon request.

Field-specific reporting

Please select the best fit for your research. If you are not sure, read the appropriate sections before making your selection.

☒ Life sciences ☐ Behavioural & social sciences ☐ Ecological, evolutionary & environmental sciences

For a reference copy of the document with all sections, see nature.com/authors/policies/ReportingSummary-flat.pdf

Life sciences study design

All studies must disclose on these points even when the disclosure is negative.

Sample size	For electrophysiology experiments (endophilin TKO characterization, rescue, endophilin BAR and ΔITSN mutants), at least three independent experiments from 3-5 litters were performed (some litters had multiple endophilin TKO animals). On average, 6 cells were recorded per genotype and per experiment.<br>For ICC, a minimum of 10 images from 3-5 independent experiments were performed and analyzed for each genotype/condition.<br>For endocytosis uptake experiments, three independent experiments were performed from three preparations (some litters had multiple endophilin TKO animals). Minimum of 10 images (oftern many more) per sample was recorded and analyzed.<br>For EM, at least 23 cells from 4 different animals and independent embeddings were recorded and analyzed.<br>For Western blot, we have pooled adrenal glands from 5 animals for each experiments. At least three independent experiments were performed and analyzed (often 4-5 exps were done).
Data exclusions	We did not exclude any data.
Replication	All experiments that are reported in this study could be and were replicated for at least three times, often more.<br>Selective datasets were also replicated by different scientists. For example, key immunocytochemisty data were done independently by Ira Milosevic in 2013, Vicky Steuber in 2014-2015 and Sindhuja Gowrisankaran in 2016-2017, and the findings were reproducible.<br>Electrophysiology reported in Figure 2 was originally done by Monika Gelker and Ira Milosevic in 2013-2014, and could be reproduced by Sindhuja Gowrisankaran in 2015. EM experiments were analyzed by two independent people, Vicky Steubler and Jana Kroll.
Randomization	N/A
Blinding	The key electrophysiology and EM experiments (Figure 2, Figure 4) were done as double-blinded experiments. Whenever feasible, analysis was done by two scientists independently (e.g. EM, electrophysiology) in a double-blinded manner.

Reporting for specific materials, systems and methods

Materials & experimental systems

n/a	Involved in the study
☐	☒ Unique biological materials
☐	☒ Antibodies
☐	☒ Eukaryotic cell lines
☒	☐ Palaeontology
☐	☒ Animals and other organisms
☒	☐ Human research participants

Methods

n/a	Involved in the study
☒	☐ ChIP-seq
☒	☐ Flow cytometry
☒	☐ MRI-based neuroimaging

Unique biological materials

Policy information about availability of materials

Obtaining unique materials	Unless detailed below, reagents and materials are available from the commercial sources (as specified in the text). The list of all reagents used is attached.<br>We have generated several plasmids specifically for this study, and details on those reagents are presented in the manuscript and below.<br>PLASMIDS: Full length endophilin 1, or endophilin 2, were cloned into lentivirus (LV) expression vector FUGW (a gift of Oliver Schlüter, European Neuroscience Institute Göttingen, Germany) containing an IRES followed by an enhanced green fluorescent protein (EGFP) to allow simultaneous yet independent expression of both proteins. Endophilin 1, and endophilin 2, were first amplified by a PCR reaction (original plasmids were described in Milosevic et al., 2011) and then inserted into the FUGW vector using XbaI and BamHI restriction enzymes. Similarly, endophilin 1-BAR and endophilin 2-BAR constructs (BAR domain and the linker sequence) were cloned by amplifying and inserting the endophilin 1-BAR and 2-BAR sequences into FUGW vector using XbaI and BamHI restriction enzymes. Endophilin 1-ΔITSN (endophilin 1 E329K+S366K - mutant that cannot bind intersectin-1; Pechstein et al, 2015) was first generated by QuikChange II Site-Directed Mutagenesis Kit (Agilent) and subsequently inserted into the FUGW vector using XbaI and BamHI restriction enzymes. All constructs were verified by sequencing and control restriction digestion.

Antibodies

Antibodies used	All antibodies used in this study are available from the commercial sources (as specified in the text).<br>Note that two antibodies for endophilin A1 (guinea pig) and endophilin A2 (rabbit) generated by Synaptic Systems (Göttingen, Germany) for the Milosevic laboratory and this study. Both antibodies were just made publicly available, and can be purchased directly from Synaptic Systems (see www.sysy.com web page for details - endophilin A1 is being sold under product number 159 004; endophilin A1 is being sold under product number 159 103).
Validation	Information regarding the validation of the commercial antibodies is available from the antibody producer, or the cited literature. In addition, as stated above, antibodies for endophilin A1 (guinea pig) and endophilin A2 (rabbit) were generated by Synaptic Systems (Göttingen, Germany) for the Milosevic laboratory and this study. Both antibodies were characterized by the Milosevic laboratory on the knock-out material as well as on endophilin-overexpressing cells, and they were then affinity purified and further characterized by Synaptic Systems. Affinity purified endophilin antibodies were then again tested by us, and used in this study.

Eukaryotic cell lines

Policy information about cell lines

Cell line source(s)	HEK-293 FT (Invitrogen/ThermoFischer Scientific - product no. R70007)
Authentication	The HEK cells were purchased directly from Invitrogen (ThermoFischer Scientific).
Mycoplasma contamination	HEK cells were tested negative for mycoplasma contamination. Primary chromaffin cells that live only 2-5 days in culture were not tested.
Commonly misidentified lines<br>(See ICLAC register)	n/a

Animals and other organisms

Policy information about studies involving animals; ARRIVE guidelines recommended for reporting animal research

Laboratory animals	Endophilin mutant mice were originally generated and described in (Milosevic et al., 2011), and are accessible from the Jackson Laboratory (strain 021573 - B6;129-Sh3gl2tm1Pdc/J; strain 021574 - B6;129-Sh3gl1tm1Pdc/J; 021575 - B6;129-Sh3gl3tm1.1Itl/J). WT (C57BL/6J) mice used as additional control were obtained from endophilin A1+/-A2+/-A3+/- mice breeding, or were purchased from ZTE Göttingen.
Wild animals	n/a
Field-collected samples	n/a
